# Rare bleeding disorders in Pakistan – a call for exploration!

**Published:** 2023

**Authors:** Hamza Khan

**Affiliations:** 1 Dr. Hamza Khan Department of Haematology, Indus Hospital and Health Network, Karachi, Pakistan

Congenital deficiencies of fibrinogen, prothrombin, factor five, factor seven, factor 10, factor 11, and combined factor five and factor eight deficiency are examples of rare bleeding diseases (RBDs).^1^ They are distributed across the world and are found in all ethnic groups. Since the majority of these are inherited in an autosomal recessive fashion, areas with a higher prevalence of consanguinity tend to have more of them.^1^,^2^ Even though they’re uncommon, RBDs can cause clinically significant bleeding, making early diagnosis and treatment initiation crucial.^1^,^2^ There is a wealth of information about various facets of these disorders in the Western and European medical literature.^1^,^3^ Pakistan is one of the countries having high rate of consanguineous marriages, so it is expected that RBDs would be more prevalent here as compared to worldwide data, but they largely remain undiagnosed in the region due to lack of specialized diagnostic facilities, lack of public awareness and low clinical suspicion in many cases.^3^,^4^ Due to these limitations, there is not much local data regarding frequency and clinical spectrum of these disorders.

Specific factor concentrates are a common treatment option for most RBDs,^1^ but their supply is scarce in our nation due to lack of resources, and even when they are, their exorbitant price puts most patients out of reach.^2^ These factors lead clinicians to use fresh-frozen plasma (FFP) and cryoprecipitate, which are not recommended as first-line therapeutic options in the majority of RBDs.^1^,^2^

Another issue is the absence of a patient registry, which prevents the correlation of patient information, the bleeding phenotype, and past clinical and treatment history amongst healthcare facilities. Pakistan is noted for having minimal health spending, and the majority of the money is allocated to the fight against infectious diseases and maternal morbidity.^5^ Establishment of specialized laboratory facilities at least in the capital city of each province along with referral centers, availability of expertise, national data-base registries, patient education and provision of services at subsidized rates would help these patients timely in their diagnosis, management and long-term follow ups. Creation of the registries would then help in the advocacy of appropriate budget allotment. All this is achievable through public-private associations with eagerness and sincerity from both the sides.
